# Tuberculosis as Viewed by a Veterinarian1Read before the Section on Public Health of the New York Academy of Medicine, November 8, 1895.

**Published:** 1895-12

**Authors:** H. D. Gill


### TUBERCULOSIS AS VIEWED BY A VETERINARIAN.[1]

By H. D. GILL, V.S.

The dangers of meat visibly and extensively affected with so-called "consumption" have been recognized ever since Moses forbade the Hebrews to eat such food, but it is only within the last few years that tuberculosis of animals has risen by rapid strides from a comparatively unknown and unstudied disease to the position of largest importance in the mind of the veterinarian and of the general public.

Several causes have contributed to this sudden rise. The certain knowledge of the cause of tuberculosis, discovered by Koch and published in 1882, the practically coincident general appreciation of the magnitude of the live-stock interest here in the United States, and the increased number of educated veterinary surgeons have all been influential in promoting the study of the disease. This study has been promoted largely, too, by the influence of the United States Government, exerted through the Bureau of Animal Industry.

The clinical work has been reinforced by pathological and bacteriological work, and at last a means of diagnosis has been discovered in tuberculin, which affords a more delicate and more trustworthy test than any hitherto used for any purpose in veterinary medicine. Armed with this new ability to recognize hidden and slightest forms of the disease, the veterinarian has discovered that herds of cattle chosen with the greatest care, kept under the most approved sanitary surroundings, considered to be in absolutely perfect health, are in many cases riddled with tuberculosis. Taking the products of these animals, both milk and flesh, to the laboratory he has discovered that the active germs of the disease are present, ready for transmission. It is on account of these incontrovertible facts, and of their immense hygienic importance, that the veterinary profession again directs your attention to them, in the hope that concerted measures may be taken to lessen these dangers.

It has been proven beyond the shadow of doubt that human

[1] Read before the Section on Public Health of the New York Academy of Medicine, November 8, 1895.

and bovine tuberculosis are identical, both being caused by the same germ (bacillus tuberculosis), which is always derived from some preëxisting case. In New York City, in the human family, over 5000 deaths from this disease occur annually. Cows are especially susceptible to this disease, and in some herds in the vicinity of New York as high as 60 per cent., and in some instances 70 per cent., of the number are affected. Tuberculosis differs from other infectious diseases in being of a slow and insidious character, and in many cases the diseased animal is to all outward appearances in perfect health. By inbreeding we can develop a predisposition in the progeny where predisposition in one or both parents already exists. The disease is transmitted from animal to animal, animal to man, and man to animal. It may pass from diseased to healthy animals in any one or more of the following ways: the virus becoming dried may be borne in the air and taken in by respiration, or it may be present on the food or on the objects which are licked by the tongue of the animal and so swallowed. The infectious matter may be derived from discharges from the mouth and nose in cases of advanced lung-disease or of disintegration of enlarged throat-glands discharging externally; from the bowels in advanced stages; from the vagina in cases of uterine tuberculosis; the milk, notably when the udder is tuberculous or the disease generalized, and possibly when there is any focus of tuberculosis in the animal; meat only when the disease is generalized; in the fœtus in cases of uterine tuberculosis.

The investigations by others and our own have shown that infection of cattle occurs in the following ways: Probably one-half of all diseased animals have been infected by inhaling the bacilli dried or suspended in the air. A large proportion of the remainder have been infected by taking tubercle bacilli into the body with food; frequently both food- and air-infection are recognized in the same animal. Animals are infected, though extremely rarely, during copulation. In such cases the disease starts in the uterus and its lymph-glands, or in the sexual organs and corresponding lymph-glands of the bull. Very rarely calves of advanced cases are born tuberculous. I know of instances where the milk from a diseased udder has been squirted upon the floor of the stable and then left to dry, to become the source of infection. I have also seen attendants who, after milking these tuberculous udders, milk healthy cows without even wiping off the adherent tuberculous milk. Such habits in attendants and the general use of milking-tubes without even a thought of cleanliness are important factors in the dissemination of tuberculosis. The infection of a healthy herd is frequently brought about by the introduction of a single tuberculous animal.

With reference to the transmission from man to animal, it may be said that such transference has undoubtedly occurred from man to swine, cats, and dogs, where these animals have eaten tuberculous sputum. It has been said that similar cases have been rarely reported as having occurred with cattle, and that statistics do not show a large proportion of tuberculous animals in a region containing many tuberculous persons than in healthy places. This, to my mind, cannot be accepted as evidence when we consider that the majority of tuberculous persons live in large cities and do not come in contact with cattle. I have in mind instances of the transmission from man to animals, through the expectoration of the attendant. There is much more danger of the transmission from man to animals by the expectoration of attendants than from animal to man by this means. The expectoration of a consumptive man is constant; bovine animals rarely throw out material raised from a disintegrating focus in their lungs, but, swallowing all such material, reinfect themselves through the intestinal tract (autoinfection).

With reference to the second possibility, the transmission of tuberculosis from animal to man, Professor Ernst's long investigation has plainly proved its possibility, and, more, its probability. The principal source of infection is through the milk from a tuberculous cow. Meat contains bacilli only when the disease becomes generalized. The majority of tuberculous cattle killed for food are worn-out milch-cows, which of necessity produce a poor quality of beef, and such beef to be made at all edible must be thoroughly cooked, thereby destroying the vitality of the bacilli. On the contrary, milk is rarely cooked, and if cooked at all is not kept at a proper temperature sufficiently long to destroy the germs or their spores; milk, therefore, to the human family is the great source of danger. The danger is enhanced by the fact that this is often the necessary and only food for infants and invalids.

The identity of bovine and human tuberculosis being placed beyond doubt by the numerous examples of contagion, by a similarity in the anatomical alterations of these diseases, and by the existence in both of the same specific bacillus, the question of consumption of the milk of tuberculous animals becomes of the greatest importance from the standpoint of public hygiene. How widespread it is can be learned, in my opinion, only when every animal has been subjected to the tuberculin-test, and until such investigation has been made the only safeguard for the milk-supply of New York is limitation to that coming from herds which have been so tested. Too great emphasis can hardly be laid on the clearly demonstrated fact that tuberculosis may exist in cattle when they present absolutely no clinical symptoms of disease. Danger is not to be especially looked for when an animal presents the classical symptoms of emaciation, cough, etc. It is when the animal is in good flesh, has a healthy skin, and nevertheless has one or more foci of disease.

While recognizing that the essential cause of tuberculosis is the bacillus, we must not ignore the fact that there are other conditions that are accessory. The first in importance is hereditary predisposition. The hereditary transmission of the disease and Baumgarten's theory of latency are particularly suggestive. The proper pabulum or soil for the development of the germ is only second in importance to the germ itself, and in taking measures to limit the diffusion of the bacillus let us not lose sight of the importance to the possible host of combating inherited weakness, of removing acquired debility, and of maintaining the system at a standard of aggressive activity. In nearly every paper read, and nearly every discussion entered into upon the subject of tuberculosis in cattle, in-breeding has been made preeminently the predisposing cause. Therefore, in conclusion, allow me to offer a suggestion along this line (leaving the balance of this subject to Professor Law, who no doubt will cover it in his paper on the eradication of the disease). If by inbreeding cattle we can develop a predisposition to disease in the progeny, where predisposition already exists in the parents, why can we not on the same principle, by judicious inbreeding with animals that we know to be absolutely free from both the predisposition and the disease, augment those characteristics in the species or family which are capable of combating or withstanding disease-elements? While the prevalent system of inbreeding is not without its dangers, I would suggest that if the same thought is exercised in regard to the freedom from predisposition to tuberculosis that is given to the individual milk- or beef-merit of the animals to be inbred, the danger of inbreeding could be reduced to a minimum, while an increased immunity might result to the progeny.

